# Neonatal microbiota-epithelial interactions that impact infection

**DOI:** 10.3389/fmicb.2022.955051

**Published:** 2022-08-25

**Authors:** Shikha Negi, Seika Hashimoto-Hill, Theresa Alenghat

**Affiliations:** Division of Immunobiology and Center for Inflammation and Tolerance, Cincinnati Children’s Hospital Medical Center, University of Cincinnati College of Medicine, Cincinnati, OH, United States

**Keywords:** neonate, intestine, epithelial, microbiota, infection

## Abstract

Despite modern therapeutic developments and prophylactic use of antibiotics during birth or in the first few months of life, enteric infections continue to be a major cause of neonatal mortality and morbidity globally. The neonatal period is characterized by initial intestinal colonization with microbiota and concurrent immune system development. It is also a sensitive window during which perturbations to the environment or host can significantly impact colonization by commensal microbes. Extensive research has demonstrated that these early life alterations to the microbiota can lead to enhanced susceptibility to enteric infections and increased systemic dissemination in newborns. Various contributing factors continue to pose challenges in prevention and control of neonatal enteric infections. These include alterations in the gut microbiota composition, impaired immune response, and effects of maternal factors. In addition, there remains limited understanding for how commensal microbes impact host-pathogen interactions in newborns. In this review, we discuss the recent recognition of initial microbiota-epithelial interactions that occur in neonates and can regulate susceptibility to intestinal infection. These studies suggest the development of neonatal prophylactic or therapeutic regimens that include boosting epithelial defense through microbiota-directed interventions.

## Introduction

Despite a steady decline in childhood mortality (Sharrow et al., 2022), enteric infections in infants continue to pose a significant health risk (Kotloff et al., 2013; Bagamian et al., 2020). Indeed, infections remain one of the major causes of mortality in both preterm and term infants (Stoll et al., 2011; Isaac et al., 2016; Troeger et al., 2018). Due to the immaturity of their developing immune system and lack of a diverse intestinal microbiota, trillions of microbes residing in the gut lumen that restrain pathogens, neonates are particularly vulnerable to infection (Miller et al., 2018). Newborns are initially dependent on epithelial defense, transfer of maternal immune factors, innate immune cell activation, and intestinal microbial colonization for protection against intestinal pathogens. Studies have emphasized that the development of the intestinal microbiota is critical for the maturation of the immune system, epithelial barrier, and colonization resistance against invading pathogens (Olszak et al., 2012; Cahenzli et al., 2013; El Aidy et al., 2013; An et al., 2014; Gensollen et al., 2016; Yu et al., 2016; Roubaud-Baudron et al., 2019; Singer et al., 2019; Travier et al., 2021). Infants become rapidly colonized during birth with the intestinal microbiota abundance, composition, and diversity continuing to mature throughout neonatal development. Further, the neonatal immune system coevolves with the intestinal microbiota, and this early window of immune cell education and development is critical for healthy immune responses later in life. Antibiotics (ABX) are commonly used to treat or prevent infections in infancy. However, early life (prenatal, perinatal, and postnatal) exposure to ABX can perturb the developing microbiota, induce epithelial barrier dysfunction and may lead to enhanced neonatal susceptibility to enteric pathogens (Schumann et al., 2005; Greenwood et al., 2014; Rogawski et al., 2015; Schulman et al., 2015; Man et al., 2019). In addition, alterations in the intestinal microbiota by ABX promote short- and long-term immunological effects extending well into adulthood. This review examines the critical role and interplay of commensal bacteria and the epithelial barrier that influence enteric infections in neonates.

## Neonatal intestinal infection

Newborns are highly susceptible to enteric pathogens, particularly during the first year of life (Liu et al., 2012; Lanata et al., 2013; Darmstadt et al., 2014; Miller et al., 2016). Most common enteric pathogens linked to neonatal morbidity and mortality include Enteropathogenic *Escherichia coli* (EPEC), *Group B streptococci* (GBS), *Listeria monocytogenes, Salmonella*, Rotavirus, and *Cryptosporidium* parasites (Abba et al., 2009; Stoll et al., 2011; Pedersen et al., 2014; Bergin et al., 2015; Shane et al., 2017; Reju et al., 2022; Yoon et al., 2022). These pathogens can gain entry orally, invade the gastrointestinal (GI) tract, and lead to further blood or systemic infections in neonates.

Enteropathogenic *E. coli* (EPEC) is responsible for many diarrhea outbreaks in newborns (Nataro and Kaper, 1998; Abba et al., 2009; Hu and Torres, 2015). EPEC infection is characterized by attaching and effacing (A/E) lesions (Cepeda-Molero et al., 2017). While adult C57BL/6 mice are resistant to human EPEC, neonatal mice exhibit age-dependent susceptibility. Pups up to 7 day-old were found to be highly susceptible, with significantly decreased susceptibility observed in 10–13 day-old (Dupont et al., 2016). The neonatal infection depended on EPEC expression of virulent factors IV bundle forming pili (BFP) and type III secretion system (T3SS), both of which are essential for A/E lesion formation and host cell invasion (Cleary et al., 2004; Iizumi et al., 2007; Galan et al., 2014). This unique susceptibility of neonates was attributed to the neonatal gut microbiota as well as epithelial responses to the pathogen that are unique to neonates, including more exaggerated upregulation of TLR-dependent genes compared to adults (Dupont et al., 2016).

Non-typhoidal *Salmonella, Salmonella enterica* subsp. *enterica*, also causes enteric diseases. *Salmonella* is classified as acid-sensitive and can be killed by the acidity in the adult stomach. However, less acidic stomach contents and faster gastric emptying of neonates favor *Salmonella* survival and small intestinal colonization (Gorden and Small, 1993; Bula-Rudas et al., 2015). Neonatal mice demonstrate a higher susceptibility to infections than adults (Zhang et al., 2014). *Salmonella* directly invades enterocytes in neonates in a T3SS dependent manner, instead of utilizing microfold cells (M-cell)-mediated uptake of pathogens observed in adults (Zhang et al., 2014). Unlike in adults, infection of C57BL/6 neonatal mice did not require streptomycin pretreatment, suggesting that characteristics of the neonatal microbiota lead to unique susceptibility (Zhang et al., 2014). A more recent study colonized adult germ-free mice with neonatal or adult cecal contents and found that microbiota from adults, but not neonates, prevented *Salmonella* colonization (Kim et al., 2017).

Group B *Streptococcus* (GBS) is an intestinal commensal that causes systemic diseases such as septicemia and meningitis in infants, but not in immunocompetent adults (Raabe and Shane, 2019). GBS gains access to the systemic circulation through invasion of the neonatal intestinal epithelium. A recent study demonstrated that immature epithelial barrier function and microbiota composition attributed neonatal susceptibility to GBS infection (Travier et al., 2021). These enteric infection studies in young mice demonstrate strong links between increased pathogen susceptibility with the neonatal microbiota and developing epithelial cell function, discussed in more detail below.

## Neonatal gut microbiota

Colonization by commensal microbes at birth is critical for the development of host immunity and defense against pathogens (Negi et al., 2019; Zheng et al., 2020; Fan and Pedersen, 2021). The microbiota composition of neonates differs from adults. In humans, *Firmicutes* and *Bacteroidetes* are the two dominant phyla in adults while *Firmicutes, Bacteroidetes, Actinobacteria*, and *Proteobacteria* predominate in term, vaginally delivered neonates (Arboleya et al., 2015; Backhed et al., 2015; Del Chierico et al., 2015; Radjabzadeh et al., 2020). Microbiota colonization begins at birth and is dominated mainly by facultative anaerobes such as *Lactobacillus, Enterococcus*, and *Streptococcus* in the initial days of life (Hansen et al., 2015; Robertson et al., 2019). Further, breastfeeding enriches the *Bifidobacterium* and *Bacteroidetes* followed by the prevalence of obligate anaerobes such as *Clostridia* in the gut. The murine commensal *Clostridia* has been shown to protect against enteric *Salmonella* infection. This protective effect was attributed to the production of metabolite succinate, though its mechanisms remain to be elucidated (Kim et al., 2017). In addition, *Bifidobacterium*-produced acetate has been reported to protect against *E. coli* infection (Fukuda et al., 2011).

The composition of the neonatal microbiota is significantly shaped by the maternal microbiota and diet (Dominguez-Bello et al., 2010; Chu et al., 2016; Asnicar et al., 2017; Shao et al., 2019; Garcia-Mantrana et al., 2020; Maher et al., 2020). There are also reports that maternal microbiota-derived factors and metabolites such as short-chain fatty acids may be sensed or passed during gestation and influence the offspring’s metabolism (Kimura et al., 2020; Pessa-Morikawa et al., 2022). Transmission of microbiota during birth is followed by early development during the first 2–3 years to ultimately more closely resemble adulthood composition (Donnet-Hughes et al., 2010; Rautava et al., 2012). This dynamic bacterial colonization inversely correlates with the occurrence of infections in neonates (Madan et al., 2012; Mai et al., 2013; Matamoros et al., 2013). Additionally, studies have demonstrated that proper microbiota colonization during this early neonatal period may impact long-term health (Torow and Hornef, 2017; Renz et al., 2018). These include murine models showing the importance of early colonization in immune development as described in the following section (Olszak et al., 2012; Al Nabhani et al., 2019). Further, human longitudinal studies have shown that neonatal microbiota compositions associate with clinical manifestations in allergy, neurodevelopment, and metabolic disorders later in childhood (Galazzo et al., 2020; Roze et al., 2020; Jian et al., 2021).

Recent advancements in metagenomic sequencing have revealed strain-level details of shared bacteria between infants and their mothers, with the mode of delivery affecting microbial exposure in early life (Asnicar et al., 2017; Ferretti et al., 2018; Shao et al., 2019). Vaginally delivered infants are often predominantly colonized by beneficial commensal bacteria such as *Bacteroides, Lactobacillus*, and *Bifidobacterium* (Hesla et al., 2014; Chu et al., 2017; Stewart et al., 2018). Interestingly, by 6 months of life, *Lactobacillus* colonization was found to be the same irrespective of delivery mode in one study (Huurre et al., 2008). In another study, *Bacteroides* were still found to be high in vaginally born infants (Stinson et al., 2018). Infants delivered *via* C-section harbor high abundance of opportunistic pathogens such as *Enterococcus* and *Enterobacter* (Jakobsson et al., 2014; Shao et al., 2019). They also exhibit delayed colonization with beneficial *Bifidobacterium* (Reyman et al., 2019).

Breast milk (BM) is often the first diet for newborns and plays a central role in shaping the neonatal microbiota (Turfkruyer and Verhasselt, 2015; Walker and Iyengar, 2015). BM contains high amounts of human milk oligosaccharides (HMOs); thus breastfed infants exhibit increased *Bifidobacterium* species that are involved in catabolism of HMOs (Backhed et al., 2015; Robertson et al., 2019). *Bifidobacterium infantis* EVC001 supplementation in term infants positively correlated with abundance of memory Treg, and negatively correlated with Th2/Th17 cytokines in the blood (Henrick et al., 2021). Further, formula-fed newborns possess more *Enterobacteriaceae* and fewer *Bifidobacterium* species (Stewart et al., 2018). Secretory IgA (sIgA) in BM also contributes to development of the neonatal microbiota, and neonates lacking sIgA have alterations in commensal communities that persist to adulthood (Rogier et al., 2014). While less characterized, viral communities also colonize the infant gut and are influenced by breastfeeding (Bushman and Liang, 2021). Furthermore, a study shows that breastfed infants harbor more temperate phages of *Bifidobacterium* or *Lactobacillus* at 4 months of age, coinciding with higher abundance of these bacteria, in comparison to formula-fed infants. On the other hand, viruses that infect human cells were less abundant in BM-fed infants’ stool, suggestive of the protective roles of BM against viral infection (Liang et al., 2020).

Antibiotics exposure both *in utero* and early postnatal life alters the gut microbiota composition of both mother and newborn, and therefore can potentially have long-lasting effects (Ohlsson and Shah, 2014; Arboleya et al., 2016; Miller et al., 2018). Exposure to commensal microbes in this early life period is critical in the development of proper immune function. For instance, GF mice harbor elevated numbers of invariant natural killer T (iNKT) cells which predispose them to chemically induced colitis. This abnormal iNKT cell phenotype can only be rescued when GF mice are recolonized on the first day of life but not in adulthood, highlighting the importance of early life immunological imprinting by the microbiota (Olszak et al., 2012). In addition, ABX-inhibition of colonization during the weaning period in mice resulted in decreased RORγt^+^ regulatory T cells and increased susceptibility to chemically induced colitis (Al Nabhani et al., 2019). Further, another study reported that neonatal mice with transient early life ABX exposure resulted in persistent microbiota alterations and increased susceptibility to enteric bacterial infection as an adult (Roubaud-Baudron et al., 2019). Therefore, these findings suggest that ABX treatment early in life may alter the microbiota in a manner that not only impacts neonatal defense, but may also increase susceptibility later in life. In preterm infants, factors such as gestational age, ABX, reduced BM consumption, environmental microbes of neonatal intensive care unit, and prolonged hospitalization contribute to the colonization with specific microbial strains such as *Enterobacter, Enterococcus, Lactobacillus, Photorhabdus*, and *Tannerella* (Ardissone et al., 2014; Collado et al., 2015).

## Intestinal epithelial cells

**FIGURE 1:**
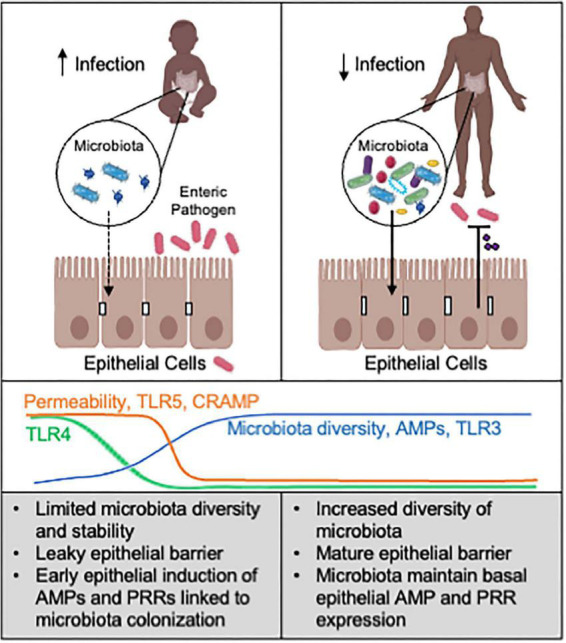
Microbiota-epithelial interactions alter susceptibility to infection. Neonates are highly susceptible to enteric pathogens, relative to older children and adults. In the neonatal intestine, a limited abundance and diversity of commensal microbes correspond with increased epithelial permeability and distinct AMP expression and microbial sensing. This less mature microbiota-epithelial relationship likely increases susceptibility to enteric infection. In contrast, the adult intestine harbors a more complex and stable microbiota that can maintain more effective epithelial defense mechanisms against pathogens. Promoting epithelial defense through microbiota-based interventions may help prevent or combat neonatal enteric infection. AMPs: antimicrobial peptides. PRRs: pattern recognition receptors. CRAMP (cathelin-related antimicrobial peptide) is AMP enriched in neonatal intestine. Line graphs are based on data from animal models. Created with Biorender.com.

Immune responses in the neonatal intestine are required to be tolerant to newly colonizing commensal bacteria while also protecting against enteric pathogens. Innate immune cells play a central role in protective immunity in the neonatal period, as adaptive immunity is still immature (Kollmann et al., 2012; Lee et al., 2019; Rudd, 2020; Westrom et al., 2020). In addition, intestinal epithelial cells (IECs) are non-hematopoietic cells that serve as the first line of defense and a key barrier to invading pathogens in both mice and humans (Allaire et al., 2018; Eshleman and Alenghat, 2021; Figure 1).

The epithelium is organized into villi and crypt structures, with stem cells residing at the base of crypts differentiating into absorptive and secretory lineages (Walthall et al., 2005; Allaire et al., 2018). Humans and pigs exhibit mature crypt-villus axes at birth while in mice, crypts become fully functional 2 weeks after birth (Walthall et al., 2005; Harper et al., 2011). Thereafter, stem cells in crypts differentiate into different lineages of IECs such as absorptive enterocytes and secretory goblet cells, Paneth cells and enteroendocrine cells (EECs) (Barker et al., 2007). The proliferation rate of epithelial stem cells in neonates is lower than in adults, which is at least partly due to elevated expression of B lymphocyte-induced maturation protein-1 (Blimp-1), a transcriptional repressor that inhibits adult-like differentiation and regulates structural and biochemical changes of IECs during the suckling-weaning transition in mice (Harper et al., 2011; Muncan et al., 2011).

### Barrier function

Intestinal epithelial cells reside directly at the interface of the developing microbiota and underlying immune system (Allaire et al., 2018; Eshleman and Alenghat, 2021). Many intracellular structures such as adherens junctions (AJ), desmosomes and tight junctions (TJ) maintains barrier integrity in both mice and humans. TJ proteins, such as claudins and occludin, seal the intercellular spaces and strictly regulate the macromolecular transport (Van Itallie et al., 2008; Suzuki et al., 2014; Ronaghan et al., 2016). Zonulin, a signaling molecule that triggers phosphorylation of epidermal growth factor receptor (EGFR) and downstream tight junction disassembly, regulates gut permeability (Sturgeon and Fasano, 2016). Fecal and serum zonulin are used as a biomarker for increased intestinal permeability in neonatal studies (Saleem et al., 2017; Kaczmarczyk et al., 2021; Sochaczewska et al., 2022). The development of intestinal barrier function occurs *in utero* in humans, with IEC tight junction proteins production observed as early as 10 weeks of gestation, followed by secretion of defensins, lysozyme, and mucin that create additional layers of a chemical and mechanical barrier (Polak-Charcon et al., 1980; Mallow et al., 1996; Buisine et al., 1998; Rumbo and Schiffrin, 2005). Epithelial barrier function is affected by oral ABX administration and alterations in microbiota composition (Schumann et al., 2005; Ma et al., 2018; Garcia et al., 2021; Sochaczewska et al., 2022). Reports differ on whether ABX increase or decrease epithelial barrier function, and this may reflect differences in the types of ABX and microbiota composition (Schumann et al., 2005; Soto et al., 2014; Zhou et al., 2020; Chaaban et al., 2022).

Maturation of barrier function continues postnatally in response to factors present in the neonatal intestine. For example, breast milk (BM) components have been described to improve epithelial barrier function in both rodent and human studies (Weaver et al., 1987; Saleem et al., 2017; Chleilat et al., 2020). Lactoferrin, an iron-binding protein, exhibits protective effects in bacterial endotoxin-induced intestinal barrier damage (Hirotani et al., 2008). Further, BM-derived transforming growth factor-beta (TGF-β) inhibits proinflammatory responses in immature human IECs and is associated with the intestinal microbiota composition in neonates (Rautava et al., 2011; Sitarik et al., 2017). BM has been shown to increase the expression of TJ protein occludin in a pediatric enteroid model (Noel et al., 2021). Furthermore, neonatal supplementation of EGF in mice prevented translocation of pathogenic bacteria by inhibiting goblet cell-associated antigen passages (GAPs) that transport luminal antigens across intestinal epithelium (Knoop et al., 2020). Unlike adults, newborn epithelium is characterized by highly endocytic vacuolated enterocytes in the distal immature small intestine that allows passage of BM immunomodulatory components across the intestine (Baba et al., 2002; Arevalo Sureda et al., 2016; Garcia et al., 2021). Further, increased expression of neonatal Fc receptor (FcRn) on epithelial cells enables transport of maternal antibodies that confer passive immunity against pathogens in neonates (Yoshida et al., 2006; Menard et al., 2010; Ben Suleiman et al., 2012).

### Antimicrobial peptides

In addition to serving as a physical barrier, IECs also produce antimicrobial peptides (AMPs), mucins, chemokines, and cytokines that prime and regulate innate and adaptive immunity. Additionally, IECs possess various membrane and cytoplasmic patterns-recognition receptors that can detect microbial stimuli (Figure 1). AMPs can inhibit microbial survival or growth, and are among one of the most evolutionarily ancient immune defense mechanisms. A diverse array of AMPs secreted by IECs provide the first line of defense against pathogens. The enzymatic AMPs such as lysozyme and phospholipase A2 (sPLA2) are mainly secreted by Paneth cells, and damage bacterial cell walls through their catalytic activities (Menard et al., 2008; Clevers and Bevins, 2013; Mukherjee and Hooper, 2015; Bel et al., 2017). Other AMPs, such as cathelicidins, C-type lectins of the regenerating islet-derived protein (reg) 3 gamma family, and defensins disrupt microbial cell walls in a non-enzymatic fashion (Ouellette, 2010; Clevers and Bevins, 2013). Defensins produced in crypts possess bactericidal activity and can promote chloride secretion that may facilitate pathogen flushing from the intestine (Baird and O’malley, 1993; Lencer et al., 1997; Ayabe et al., 2000).

Paneth cells, major AMP producers in the small intestinal crypt, develop prenatally at 13 weeks of gestation in humans and postnatally within 2 weeks in mice (Rumbo and Schiffrin, 2005; Menard et al., 2008; Heida et al., 2016). Accordingly, a murine study showed gradual upregulation of Paneth cell specific AMPs, including defensins and lysozyme, by small intestinal IECs in the first 4 weeks of life (Menard et al., 2008). Interestingly, the same study observed neonate-specific expression of cathelin-related antimicrobial peptide (CRAMP) in IECs that likely regulate antibacterial defense and commensal colonization in early life (Menard et al., 2008). In human fetal intestine, Paneth cells and AMPs such as defensin and lysozyme expression have been reported (Heida et al., 2016). Expression of other AMPs whose expression can be induced by microbiota is postulated to occur postnatally (Kai-Larsen et al., 2007). The unique makeup of AMPs in newborns may permit initial commensal establishment (Darnaud et al., 2018; Fulde et al., 2018; Liang et al., 2022) but may be insufficient for defense against early enteric pathogens (Figure 1).

### Epithelial sensing of microbes

Intestinal epithelial cells of neonatal mice exhibit variable expression of the pattern recognition receptors (PRRs) that recognize conserved structures on beneficial commensals and harmful pathogens (Stadnyk, 2002). These PRRs activate signaling cascades in IECs that result in induction of AMPs, as well as epithelial cytokine production (Peterson and Artis, 2014). Immediately after birth, epithelial sensing of lipopolysaccharides (LPS) *via* Toll-like receptor (TLR)4 triggers establishment of LPS tolerance through dampening of TLR4 signaling through repression of interleukin 1 receptor associated kinase 1 (IRAK1) (Lotz et al., 2006). This LPS tolerance in the intestine is crucial for promoting microbiota colonization and inhibiting inflammatory responses as lack of endotoxin resistance leads to bacterial-induced IEC apoptosis and loss of barrier integrity (Lotz et al., 2006; Chassin et al., 2010). Additionally, TLR2 which senses bacterial lipoproteins is expressed in neonatal IECs. TLR2 and TLR4 overexpression was reported in a premature rat necrotizing enterocolitis model and positively correlated with disease severity (Le Mandat Schultz et al., 2007). Further, neonatal IECs exhibit >100-fold higher expression of TLR5, a PRR that recognizes bacterial flagellin, relative to IECs from adults (Fulde et al., 2018). The high expression of TLR5 in neonates may not contribute to protection against pathogens as neonatal TLR5 knockout mice did not exhibit increased susceptibility to *Salmonella* infection. Instead, TLR5 was essential in the colonization of symbiotic microbiota, partly through upregulation of the AMP Reg3γ (Fulde et al., 2018). In contrast, expression of TLR3, which detects viral dsRNA, was >20-fold lower in neonatal mice compared to adults. Interestingly, epithelial TLR3 expression inversely correlated to rotavirus infection (Pott et al., 2012), suggesting insufficient epithelial TLR3 expression in neonates may underlie their unique susceptibility to this pathogen.

In summary, dynamic epithelial regulation of barrier function, AMP expression, and microbial sensing in the intestine seem to each be critical factors underlying neonatal susceptibility to enteric infection (Figure 1).

## Therapeutic approaches and future directions

Given the strong link between neonatal microbiota and enteric infection, microbiota-based therapies may be effective in reducing neonatal morbidity and mortality caused by enteric infection. Probiotics are live microbial supplements that have the potential to impact the host microbiota and suppress pathogenic outgrowth in the intestine (Gerritsen et al., 2011; Quigley, 2019). Probiotics may be used alone or in combination with prebiotics, which include dietary fibers that can promote expansion of beneficial microbial species (Quigley, 2019). *Lactobacillus* probiotics have shown therapeutic benefits in infants and young children with enteric infection, although these effects have not been observed universally (Szajewska and Mrukowicz, 2001; Van Niel et al., 2002; Szajewska et al., 2007; Freedman et al., 2018; Schnadower et al., 2018). A combination of *Lactobacillus plantarum* and a prebiotic fructooligosaccharide may provide defense against sepsis in early- and full-term infants based on a large-scale randomized placebo-controlled study (Panigrahi et al., 2017). However, effects of probiotics for sepsis in preterm infants have varied (Rao et al., 2016; Morgan et al., 2020). Although considered rare, probiotic sepsis associated with live strains, particularly in preterm infants with immature barrier functions, could present potential adverse effects (Chiang et al., 2021; Kulkarni et al., 2022). Thus, there is clear need for further investigation into neonatal host-microbiota interactions to develop efficacious and safe microbiota-based therapeutic approaches.

The neonatal period represents a unique window of opportunity for guiding improved microbiota-based strategies that could have a greater impact on infection prevention. Thus, further taxonomic, and functional characterization of neonatal microbiota in relation to maturity, diet, and age would aid development of such therapeutic and prophylactic approaches. Preventing the disruption of initial microbial colonization while strengthening neonatal intestinal immunity can impart protection against enteric pathogens. Additionally, current studies linking effects of breastfeeding and microbiota warrant continued exploration. Given the inefficiency of neonatal adaptive immunity, microbiota-targeted therapies, including during the prenatal period, may allow induction of epithelial defenses that can boost early innate defense. Further investigation into microbiota-based strategies to enhance basal epithelial antimicrobial and barrier responses may guide improved strategies for reducing neonatal morbidity and mortality to enteric infection.

## Author contributions

SN, SH-H, and TA wrote the manuscript. All authors contributed to the article and approved the submitted version.
